# Identification of Novel Stress Granule Components That Are Involved in Nuclear Transport

**DOI:** 10.1371/journal.pone.0068356

**Published:** 2013-06-27

**Authors:** Hicham Mahboubi, Evangeline Seganathy, Dekun Kong, Ursula Stochaj

**Affiliations:** Department of Physiology, McGill University, Montreal, Quebec, Canada; University of British Columbia, Canada

## Abstract

**Background:**

Importin-α1 belongs to a subfamily of nuclear transport adaptors and participates in diverse cellular functions. Best understood for its role in protein transport, importin-α1 also contributes to other biological processes. For instance, arsenite treatment causes importin-α1 to associate with cytoplasmic stress granules (SGs) in mammalian cells. These stress-induced compartments contain translationally arrested mRNAs, small ribosomal subunits and numerous proteins involved in mRNA transport and metabolism. At present, it is not known whether members of all three importin-α subfamilies locate to SGs in response to stress.

**Results:**

Here, we demonstrate that the oxidant diethyl maleate (DEM), arsenite and heat shock, promote the formation of cytoplasmic SGs that contain nuclear transport factors. Specifically, importin-α1, α4 and α5, which belong to distinct subfamilies, and importin-β1 were targeted by all of these stressors to cytoplasmic SGs, but not to P-bodies. Importin-α family members have been implicated in transcriptional regulation, which prompted us to analyze their ability to interact with poly(A)-RNA in growing cells. Our studies show that importin-α1, but not α4, α5, importin-β1 or CAS, associated with poly(A)-RNA under nonstress conditions. Notably, this interaction was significantly reduced when cells were treated with DEM. Additional studies suggest that importin-α1 is likely connected to poly(A)-RNA through an indirect interaction, as the adaptor did not bind homopolymer RNA specifically *in vitro.*

**Significance:**

Our studies establish that members of three importin-α subfamilies are *bona fide* SG components under different stress conditions. Furthermore, importin-α1 is unique in its ability to interact with poly(A)-RNA in a stress-dependent fashion, and *in vitro* experiments indicate that this association is indirect. Collectively, our data emphasize that nuclear transport factors participate in a growing number of cellular activities that are modulated by stress.

## Introduction

The appropriate response to environmental or disease-related stress is essential for the survival of all organisms. To cope with these challenges, the formation of cytoplasmic stress granules (SGs) is one of the conserved strategies in eukaryotes [1]. Various forms of stress, including heat shock and arsenite, inhibit translation and trigger the assembly of SGs (reviewed in [2]), granular compartments that contain translationally arrested mRNAs and RNA-binding proteins [3]. Aside from poly(A)-RNA, several proteins locate to SGs under various stress conditions and therefore serve as common markers for this compartment [4], [5]. Such markers include the RNA binding proteins G3BP1 and HuR [6], [7].

Although SGs appear as well-defined cytoplasmic compartments, they are highly dynamic; moreover, their composition can differ according to the stressor and cell type [3], [5], [8].

SGs are not the only cytoplasmic RNA/protein foci; for example, under normal and stress conditions processing bodies (PBs) are present in the cytoplasm, where they can act as mRNA silencing sites [9]. PBs are spatially and functionally connected to SGs [10] and frequently juxtaposed to SGs when cells are stressed [10]. Recent studies suggest links between SGs, PBs and the nuclear transport apparatus, since transport factors of the importin-α adaptor or importin-β carrier families have been detected in both compartments ([11], see below). Notably, these transport factors are highly dynamic and affected by physiological and environmental cues, a property they share with SGs and PBs.

Among nuclear transport factors, the importin-α family participates in nuclear transport, spindle formation, ubiquitin-mediated protein degradation and the reassembly of nuclear envelopes after mitosis [12], [13], [14]. As a transport adaptor, importin-α recognizes the nuclear localization signal (NLS) on cargo proteins and forms a ternary complex with importin-β1 that translocates into the nucleus [15], [16]. Following cargo delivery to the nucleoplasm, importin-α is returned to the cytoplasm by the importin-β-like carrier CAS (cellular apoptosis susceptibility protein; reviewed in [17]).

Seven importin-α family members have been identified in humans so far [18]; according to their sequence similarity, they are classified into three subfamilies: α1/NPI1-like (importin-α5, α6, α7), α2/Rch1-like (importin-α1, α8), and α3/Qip1-like (importin-α3, α4) [19], [20], [21], [22]. Independent of the subfamily, all importin-α proteins share structural features, including armadillo repeats [23] and basic residues in the N-terminal portion that interact with importin-β1 [24]. Despite these similarities and some functional redundancy, the three importin-α classes may differ in their mode of NLS recognition and cargo preference [20], [25]. The importance of distinct importin-α proteins is emphasized by isoform switch during differentiation and development (reviewed in [26], [27]), as observed in the nematode *Caenorhabditis elegans, Drosophila* and other model systems [28], [29], [30], [31], [32].

The best-studied member of the importin-α family is importin-α1 (karyopherin-α2, *KPNA2*); this adaptor belongs to the α2 subfamily and shuttles between the nucleus and cytoplasm under normal growth conditions. At steady-state, the protein locates predominantly to the cytoplasm and nuclear envelope [33], but stress alters this distribution. For instance, heat shock accumulates importin-α1 in nuclei [34], [35] through the increase in nucleoplasmic retention and the reduction of nuclear exit [34]. Additional, and possibly unique, biological functions for importin-α1 have emerged recently. For example, importin-α1 is implicated in the transcriptional regulation of hydrogen peroxide treated cells [36] and the proliferation of human breast cancer cells [37]; importin-α1 has also been detected in SGs [38]. Unlike importin-α1, the subcellular distribution and biological functions of other importin-α proteins are much less characterized. Among the poorly understood family members is importin-α4 (hSRP1γ), which is part of the α3 subfamily. The importin-α4 coding region was initially isolated from a HeLa cDNA library, and the protein is highly abundant in brain and skeletal muscle cells [39]. On the other hand, importin-α5 (α1 subfamily) regulates proliferation in HeLa cells [40], binds to the transcription regulator STAT1 and interacts with a variety of virus proteins [41], [42].

While the importin-α family encompasses seven highly related members, the importin-β family is far more complex. Among the more than 20 importin-β proteins in mammals, some are specialized for nuclear export or import, whereas others promote transport in both directions [43], [44], [45]. Many importin-β carriers bind the NLS directly (reviewed in [46]); however, importin-β1 also associates with cargo through the adaptor importin-α. This adaptor function and their nuclear export by CAS are features shared by all three importin-α subfamilies. Like importin-α1, CAS plays a role as transcriptional regulator and is thus linked to RNA metabolism [36], [47]. Interestingly, several members of the importin-β family have been detected in SGs or PBs. Whereas transportin-1 located to both SGs and PBs upon arsenite treatment, importin-β1 associated with SGs, and importin-13 with PBs [11]. On the basis of their presence in SGs, it was speculated that nuclear transport factors participate in SG assembly [48].

Although there is a growing body of information on importin-α and β isoforms, the full spectrum of their biological function is far from being understood. This includes the response to environmental changes and prompted us to examine the effect of different stressors on importin-α1, α4, α5 as well as importin-β1 and CAS.

With our current contribution, we provide several lines of evidence that connect nuclear transport factors to cytoplasmic SGs and RNA metabolism. In particular, we defined the oxidant-induced changes as they relate to transport factor localization and RNA-association. Our work demonstrates that not only importin-α1, but also importin-α4, α5 and importin-β1, but not CAS, locate to SGs under different stress conditions. By contrast, none of the transport factors was detected in PBs under normal or stress conditions. Moreover, we identified importin-α1 as a novel protein that associates with poly(A)-RNA *in vivo* in a stress-controlled fashion. This distinguishes importin-α1 from other transport adaptors, importin-β1 and CAS. Further characterization of the importin-α1/poly(A)-RNA interaction with *in vitro* homopolymer binding assays indicate that the protein does not bind RNA directly. Taken together, our results provide new links between the nucleocytoplasmic transport machinery, RNA metabolism and the stress response.

## Materials and Methods

### Cell Culture and Stress Exposure

HeLa S3 cells were grown in Dulbecco’s modified eagle medium (DMEM) containing antibiotics and 8% fetal bovine serum. Cultures were maintained in a 37°C incubator with 5% CO_2_. To induce the formation of SGs, established conditions were used; oxidative stress was generated with 0.5 mM sodium arsenite for 30 min [49] and controls were incubated with water. Alternatively, HeLa cells were treated with 2 mM diethyl maleate (DEM, [33]) or ethanol (control) for 4 hours. Heat shock was performed for 1.5 hours at 45.5°C; a 1.5 hour heat exposure was selected because it was a reliable condition for SG induction.

### Immunofluorescent Staining

Cells were grown to 70% confluency on poly-lysine coated cover slips. After treatment, immunofluorescent staining was performed essentially as published [50]. The following antibodies and dilutions were used: importin-α1 (1∶400; Santa Cruz, sc-6917), importin-α4 (1∶2,000; gift from Dr. K. Weis), importin-α5 (1∶500; Zymed), importin-β1 (1∶1,000; sc-11376); CAS (1∶1,000; sc-1708), HuR (1∶2,000 sc-5261; 1∶1,000, Millipore 07-1735), G3BP1 (1∶1,000, BD Biosciences; 1∶2,000; kindly provided by Dr. I. Gallouzi), Dcp1 (1∶200, sc-100706; 1∶800, gift from Dr. I. Gallouzi). In brief, cells were fixed with 3.7% formaldehyde in PBS, permeabilized with 0.1% Triton X-100 in PBS and blocked in PBS/2 mg/ml bovine serum albumin/0.05% Tween-20 (PBS/BSA/Tween). Alternatively, blocking and antibody incubations were carried out with PBS/0.05% Tween/5% fetal bovine serum. Samples were incubated overnight with primary antibodies diluted in blocking solution and washed several times. FITC- and Cy3-labeled secondary antibodies (diluted 1∶500; Jackson ImmunoResearch) were added for 2 hours. Samples were washed, nuclei stained with 1 µg/ml 4′, 6-diamidino-2-phenylindole (DAPI) and cover slips mounted. Images were acquired with a Zeiss LSM510 confocal microscope in the multi-track mode, using appropriate filter settings to minimize cross-talk between the channels. Image processing was performed in Adobe Photoshop CS4. To monitor non-specific staining, pre-immune serum and isotype controls were tested under identical conditions (Fig. S1). In these experiments, little or no background staining was observed.

### Quantification of SG Localization

The association of importin-α family members or importin-β1 with SGs was evaluated using the SG marker HuR as a reference. For each transporter three independent experiments were performed for every stressor. SGs were identified based on HuR and the presence of individual nuclear transporter was determined. To this end, 30 SG-positive cells were randomly selected, and all of their SGs were scored. Data for importin-α1, α4, α5 or importin-β1 are depicted in the results section.

### Oligo-(dT) Binding Assay

HeLa cells were grown to 70% confluency and treated with ethanol (control) or DEM. Cell extracts were prepared in binding buffer (100 mM KCl, 25 mM Tris-HCl, pH****7.4, 0.1% Triton-X, 10 mM EDTA, 10 mM vanadyl adenosine complex), containing a protease inhibitor cocktail [7], [51]. Samples were passed through a 26.5 gauge needle 4 times and incubated with DNase at 37°C for 15 min. After addition of DTT (5 mM final concentration) samples were centrifuged 1 min at 12,000 rpm (microfuge). Supernatants (starting material) were incubated with 300 µl Oligo-(dT)-cellulose (BioShop, equilibrated in binding buffer) for 30 min at room temperature. The resin was collected by centrifugation and washed twice in binding buffer. Bound proteins were eluted in gel sample buffer and analyzed by Western blotting.

### Generation of Homopolymers for *in vitro* RNA Binding

Poly(A), poly(U), poly(C) or poly(G) homopolymers were coupled to BrCN-activated sepharose (Santa Cruz Biotechnology) following standard procedures. To monitor unspecific binding to the resin, the same procedure was applied to BrCN-activated sepharose, but without addition of a homopolymer.

### *In vitro* RNA Homopolymer Binding Assay

His6-tagged importin-α1 or poly(A)-binding protein (plasmid encoding poly(A)-binding protein was kindly provided by Dr. N. Sonenberg) were synthesized in *E. coli* and purified with Ni-NTA agarose (Qiagen). Purified proteins were dialyzed against 20 mM Tris HCl, pH 7.4, 150 mM NaCl and 2.5 mM MgCl_2_ and employed for *in vitro* RNA homopolymer binding, essentially as described [52], [53]. To reduce nonspecific binding, homopolymers were pre-incubated overnight with 500 µl binding buffer (BB; 20 mM Tris HCl, pH 7.4, 150 mM NaCl, 2.5 mM MgCl_2_, 0.5% NP-40, 1 mM vanadyl adenosine complex, protease inhibitors) containing 200 µg BSA/ml. In control experiments, homopolymers were digested with micrococcal nuclease (1,200 Kunitz units per digest) for 1 h at 37°C and then blocked overnight with BSA as described above. Blocked resin (poly(A), poly(U), poly(C), poly(G)-sepharose or non-conjugated sepharose) was incubated for 10 min at room temperature with 5 µg purified protein and 100 µg BSA in 500 µl BB. Unbound material was removed by centrifugation (microfuge, 1 min, 13,000 rpm**)**, and resins were washed once with BB containing 2 mg/ml heparin (10 min, room temperature) and once with BB/heparin/1 M NaCl (10 min, room temperature). Bound protein was eluted with twofold concentrated gel sample buffer by incubation at 95°C for 15 min and the resin was removed by centrifugation (5 min, 13,000 rpm, microfuge). Eluted and unbound material was analyzed side-by-side by Western blotting with antibodies against importin-α1 or the His6-tag.

### Western Blotting

Western blotting and ECL with HRP-conjugated secondary antibodies followed standard procedures [33]. Primary antibodies were used at the dilutions described for immunostaining. None of the proteins were detected by pre-immune or isotype control antibodies (Fig. S2). Poly(A)-binding protein was visualized with antibodies against the His-tag (Affinity Bioreagents, PA1-983A, diluted 1∶500).

### Statistics

For Western blot analyses results were normalized to controls; three independent experiments were carried out for each treatment. Significant differences were identified by Student’s *t-test*, all data are shown as averages+SEM.

## Results

### DEM-induced Oxidative Stress Targets Importin-α1 to Stress Granules

**Figure 1:**
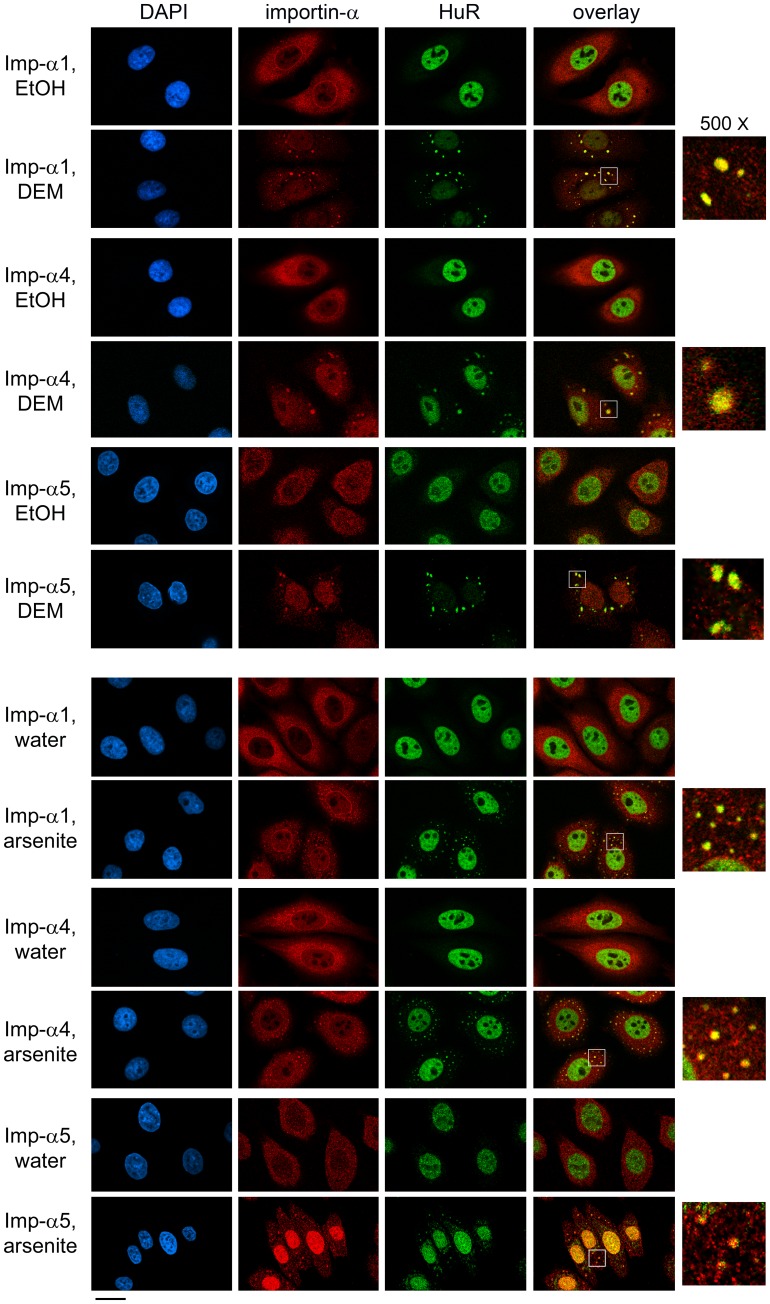
Different forms of oxidative stress target importin-α family members to SGs. HeLa cells were exposed to DEM or arsenite. Importin-α1, α4 and α5 as well as the SG marker HuR were located by indirect immunofluorescence. Nuclei were stained with DAPI; size bar is 20 µm. Co-localization of HuR and importin-α family members is shown for the selected regions (white square in overlay panel) at a magnification of 500×.

**Figure 2:**
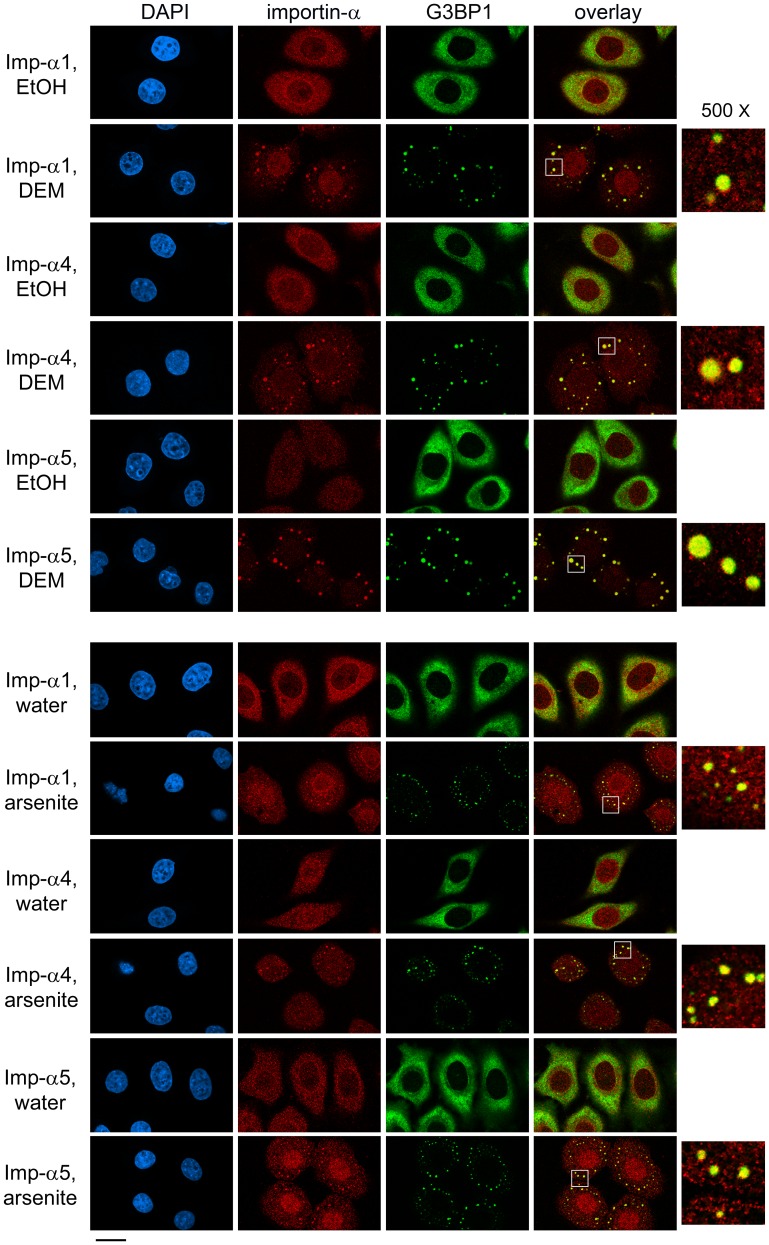
Oxidants DEM and arsenite promote the association of importin-α family members with SGs. Experiments shown in Fig. 1 were performed with the SG marker G3BP1. Nuclei were detected with DAPI; size bar is 20 µm. Co-localization of G3BP1 and members of the importin-α family can be seen in the selected regions (white square in overlay panel) at a magnification of 500×.

We and others have shown previously that stress interferes with nuclear import, in part by retaining importin-α1 in the nucleus [33], [34], [35], [54]. More recently, Fujimura et al. [38] demonstrated that sodium arsenite, an inducer of oxidative stress, triggers the association of importin-α1 with cytoplasmic SGs. Since the composition of SGs depends on the stressor, it was not known whether other oxidants also cause the translocation of importin-α1 to SGs. We therefore determined how DEM, a compound that generates oxidative stress by depleting intracellular glutathione [55], affects the distribution of importin-α1 in the cytoplasm. Fig. 1 demonstrates that in response to DEM treatment importin-α1 not only accumulates in the nucleus, but also associates with SGs, as it co-localizes with the SG marker protein HuR (Fig. 1). In independent experiments, the same results were also observed when the SG nucleating protein G3BP1was used as a reference (Fig. 2).

### Importin-α4 and α5 are Novel Constituents of SGs

**Figure 3:**
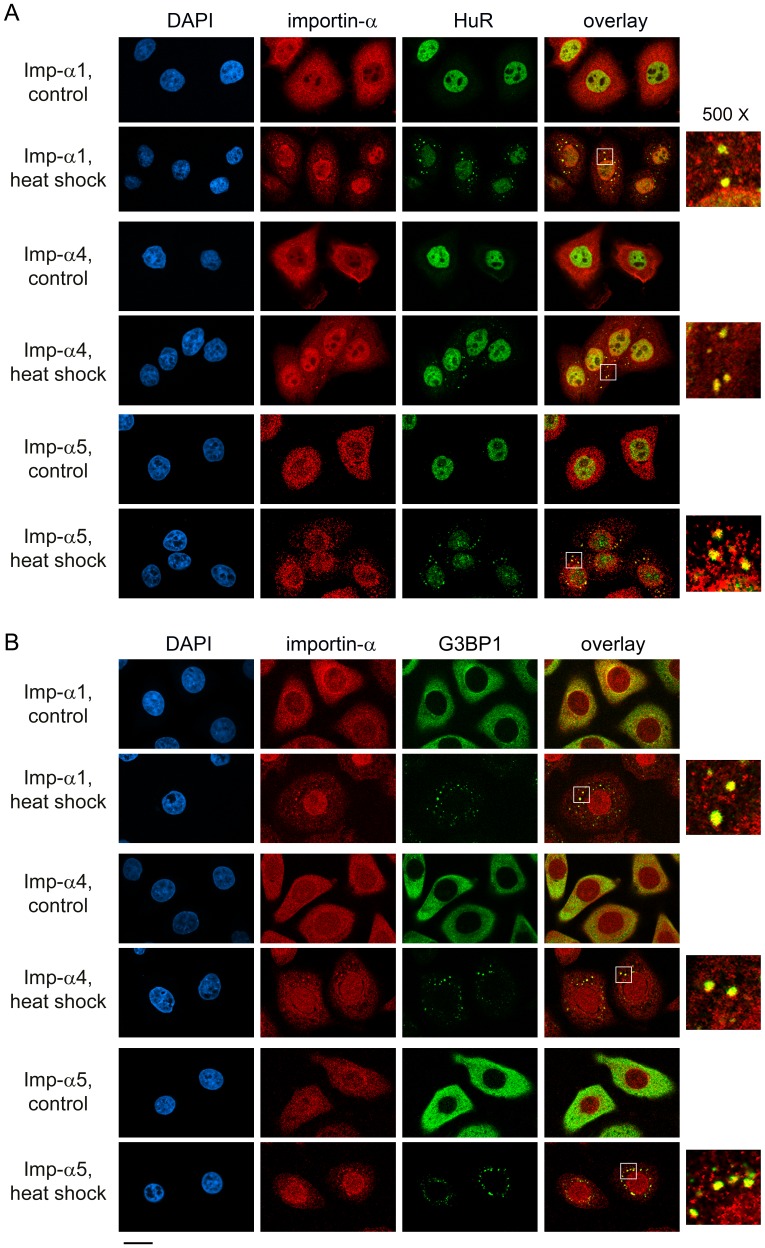
Heat shock induces the association of importin-α1, α4 and α5 with SGs. Following a 1.5-hour heat shock, the distribution of importin-α proteins and SG marker HuR (A) or G3BP1 (B) was determined. DAPI was used to stain DNA; size bar is 20 µm. The area demarcated by the white square shows a 500× magnified view of SGs.

Importin-α1 belongs to the α2 subfamily, and little is known about the stress-dependent changes of other subfamilies. We therefore extended our studies to importin-α4 (*KPNA3*) and α5 (*KPNA1*), which are representatives of subfamilies α3 and α1, respectively. To test whether importin-α4 or α5 is present in DEM-induced SGs, we located these proteins by imunocytochemistry, with two different SG markers, HuR (Fig. 1) and G3BP1 (Fig. 2). A comparison of the steady-state distribution of the different family members revealed that in unstressed cells importin-α1, α4 and α5 were predominantly cytoplasmic and concentrated at the nuclear envelope (Fig. 1, 2, 3). However, upon incubation with DEM, importin-α4 and α5 accumulated in nuclei and SGs; these results were consistently obtained, with either HuR or G3BP1 as SG marker (Fig. 1, 2). Taken together, DEM treatment is a reliable method to promote the formation of SGs; such DEM-induced SGs contain members of the three importin-α subfamilies.

### Different Stressors Target Importin-α Family Members to SGs

Although SGs assemble when translation is arrested, their composition may vary, as it is dictated by the type of stress. To address this point for importin-α isoforms, we monitored the impact of arsenite (Fig. 1, 2) and heat shock (Fig. 3A, B), following protocols that are routinely used to induce SGs [38], [56]. Like DEM, these treatments led to the formation of SGs, although the granules were smaller in size for arsenite and present in fewer cells for heat shock. Despite these differences, importin-α1, α4 and α5 relocated to SGs, which we observed when HuR or G3BP1 was used as a SG marker. Thus, we established that diverse forms of stress lead to the recruitment of different importin-α family members to SGs.

### Importin-β1, but not CAS, Associates with SGs under Various Stress Conditions

**Figure 4:**
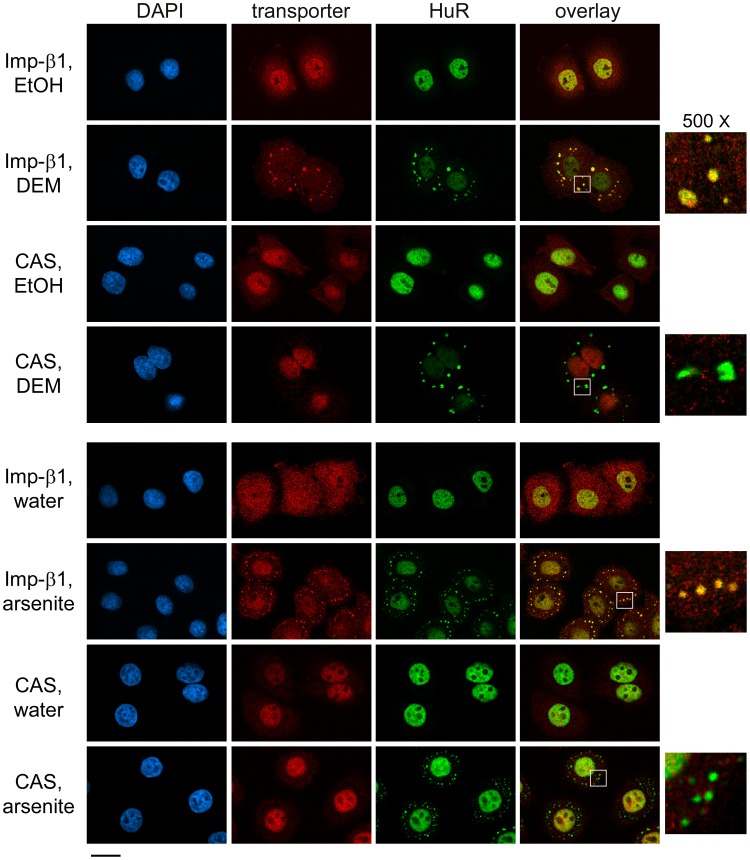
Importin-β1, but not CAS, binds to SGs in response to oxidative stress. HeLa cells treated with DEM or arsenite were stained with antibodies against the carrier importin-β1 or CAS. Co-staining was performed with the SG marker HuR; nuclei were detected with DAPI. Size bar is 20 µm; SG-containing areas (white squares) are magnified 500×.

**Figure 5:**
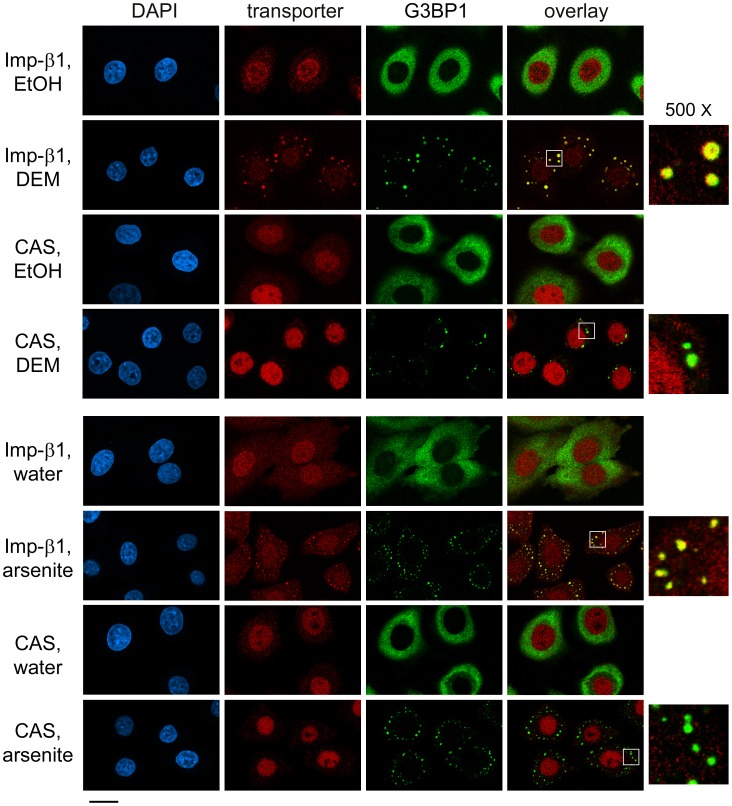
Oxidant-induced SGs contain importin-β1, but not CAS. The selective association of importin-β1 with SGs under oxidative stress conditions was examined with the SG marker G3BP1. DAPI was used to identify nuclei; size bar is 20 µm; SG-containing regions (white squares) are shown at 500× magnification.

**Figure 6:**
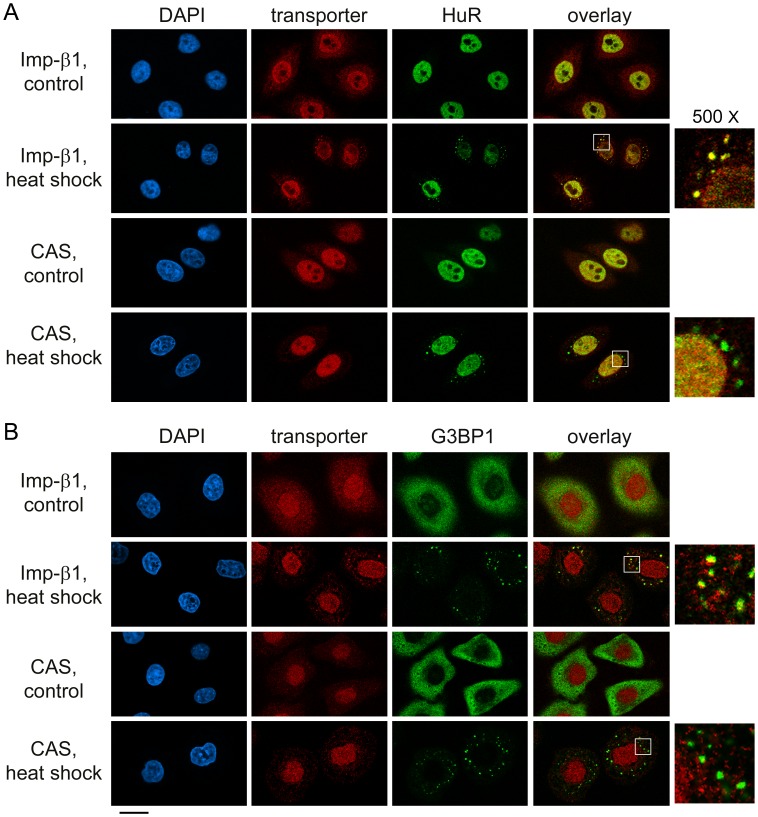
Heat shock targets importin-β1, but not CAS, to SGs. In heat-shocked HeLa cells, importin-β1, CAS and HuR (A) or G3BP1 (B) were detected by indirect immunofluorescence. DNA was stained with DAPI; size bar is 20 µm. Magnified views (500×) of SG-containing regions are depicted for stressed cells.

Nucleocytoplasmic transport mediated by importin-α requires that the adaptor interacts with importin-β1 during import to move cargo into the nucleus. On the other hand, CAS serves as the nuclear exporter for importin-α. Thus, importin-β1 and CAS, both members of the importin-β family, interact directly with different importin-α isoforms. Importin-β1 had been detected in arsenite-induced SGs [11]; however, to the best of our knowledge the impact of other stressors has not been determined. Moreover, it is not known whether CAS associates with SGs under stress conditions. Data depicted in Fig. 4, 5, and 6 reveal that importin-β1 is present in SGs following DEM, arsenite or heat treatment. As described for importin-α above, DEM produced a marked relocation of importin-β1 to SGs (Fig. 4), and the carrier was also detected in heat-induced SGs (Fig. 6). By contrast, none of the stressors caused CAS to concentrate in SGs; these results were independent of the SG marker, as they were obtained with HuR or G3BP1 as a reference. Collectively, our experiments demonstrate that the stressors which recruit importin-α proteins to SGs also promote the association of importin-β1, but not CAS, with this compartment.

### Importin-α1, α4, α5 and Importin-β1 are *bona fide* SG Components

**Figure 7:**
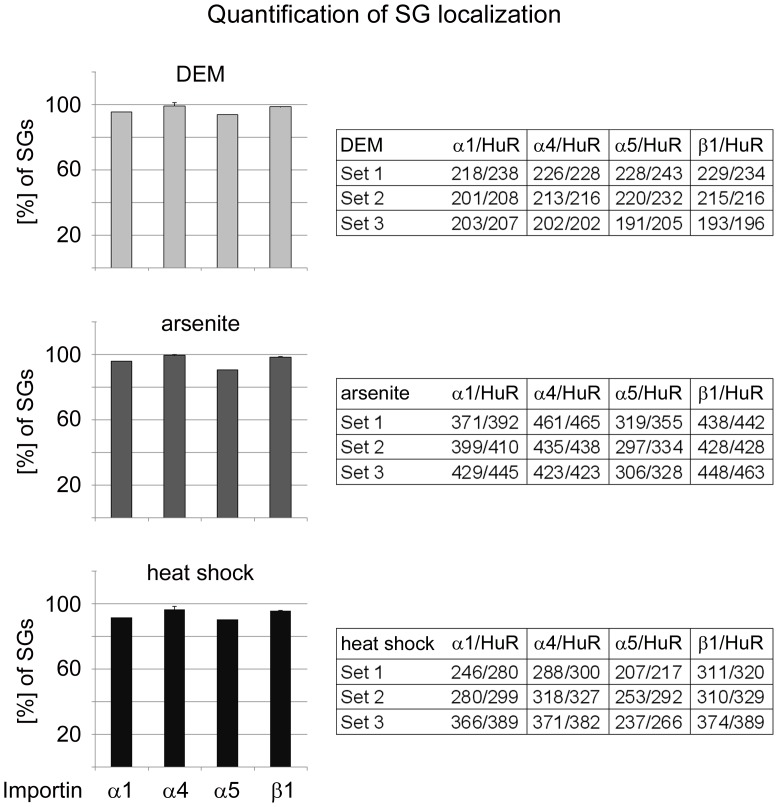
Quantification of the SG association for importin-α1, α4, α5 and importin-β1. HeLa cells were incubated with DEM, arsenite or heat shock and stained with antibodies against importin-α1, α4, α5, or importin-β1. SGs were identified with the marker protein HuR, and 30 SG-containing cells were evaluated for every treatment. Each individual SG was scored for the presence of importin-α1, α4, α5 or importin-β1. A single data point represents the average of three independent experiments+SEM. The tables depict the number of SGs that were positive for the importin analyzed/number of SGs identified with HuR; results are shown for each individual experiment. Note that more than 90% of SGs were positive for the examined members of the importin-α family or importin-β1.

To better define the association of nuclear transport factors with SGs, cells were stressed with DEM, arsenite or heat and SGs were demarcated with the marker protein HuR. SGs were then assessed for the presence of nuclear transport factors in three independent experiments for each stressor. As shown in Fig. 7, importin-α1, α4, α5 and importin-β1 were present on average in more than 90% of the SGs, with little variability between experiments. Moreover, similar results were obtained for DEM, arsenite and heat shock, suggesting that importin-α1, α4 and α5 represent novel genuine markers for cytoplasmic SGs under diverse stress conditions.

### Importin-α Isoforms and Importin-β1 Bind Specifically to SGs, but not PBs

**Figure 8:**
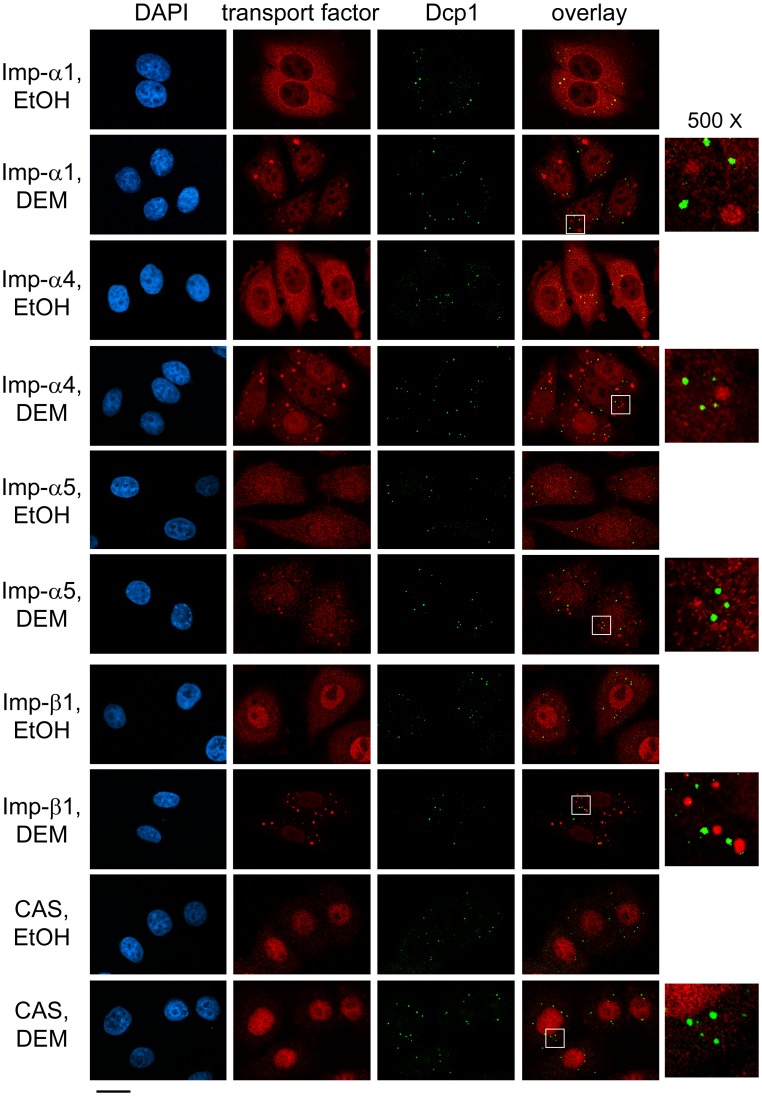
Transport factors importin-α1, α4, α5, importin-β1 and CAS do not associate with PBs under normal or stress conditions. Transport factors and the PB marker Dcp1 were detected in HeLa cells treated with DEM as described for Fig. 1. Nuclei were stained with DAPI; size bar is 20 µm. Selected PB-containing regions are shown at a magnification of 500×.

In arsenite-treated cells, transportin-1 localizes to both SGs and PBs, while importin-13 is restricted to PBs [11]. This prompted us to examine whether the transport factors analyzed here are present in PBs. Dcp1, a protein involved in mRNA decapping and degradation, was used as an established PB marker. Fig. 8 demonstrates that importin-α1, α4 and α5 were not present in PBs, neither under normal nor under stress conditions. Accordingly, in response to different stressors, the three transport adaptors were selectively recruited to SGs. Similar results were obtained for importin-β1, which was not detected in PBs (Fig. 8 and [11]), while CAS was absent from both SGs and PBs.

### Importin-α1 Associates with Poly(A)-RNA *in vivo* in a Stress-dependent Fashion

**Figure 9:**
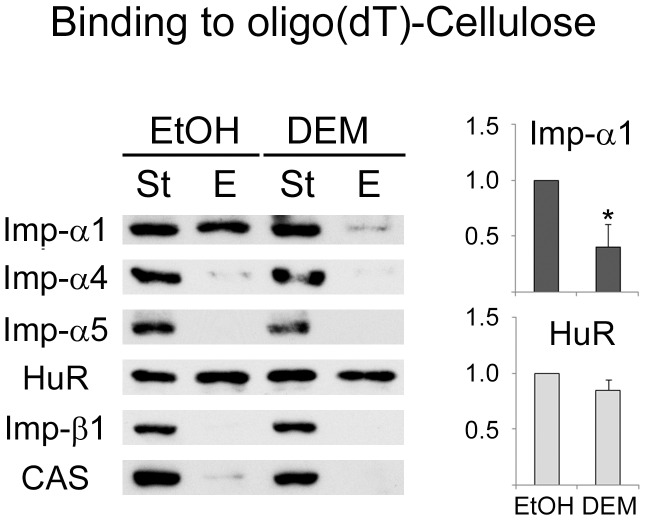
The association of importin-α1 with poly(A)-RNA in growing cells is regulated by stress. Crude extracts prepared for controls (ethanol, EtOH) and DEM-treated cells were incubated with oligo-(dT)-cellulose as described in Materials and methods. Aliquots of the start (St, 10%) and eluted (E, 100%) material were analyzed by Western blotting with different antibodies as indicated. The relative binding was calculated for importin-α1 and HuR; results were normalized to control samples. Data for three independent experiments are shown as average +SEM. Student’s *t-test* was applied to identify significant differences between control and DEM-treated samples; _*,_ p<0.05. There was no significant change for HuR.

The mechanisms that target importin-α1 to SGs are currently not understood. In principle, the recruitment to SGs could be mediated by mRNA, protein or both. We therefore examined the possibility that transport factors bind RNA under normal or stress conditions. Our studies focused on DEM treatment, because this oxidant reliably leads to the assembly of prominent SGs. Crude extracts generated from control and DEM-treated cells were incubated with oligo-(dT) cellulose, and bound material was analyzed by Western blotting. In these experiments, importin-α1 associated with the oligo-(dT) resin, suggesting that the protein interacts with poly(A)-RNA (Fig. 9). By contrast, negligible or no binding was detected for importin-α4, α5, β1 or CAS. In control experiments, HuR, a protein known to bind poly(A)-RNA [7], was efficiently pulled down with the resin (Fig. 9). Interestingly, the interaction between importin-α1 and poly(A)-RNA was strongly affected by oxidative stress. Although this adaptor bound poly(A)-RNA in control cells, the association was reduced significantly in DEM-treated cells. On the other hand, the binding of HuR was only slightly diminished by DEM. Taken together, our studies show, for the first time, that the nuclear transport factor importin-α1, but not other transport components analyzed here, associates with poly(A)-RNA. Notably, this interaction is sensitive to stress.

### *In vitro* Binding of Importin-α1 to RNA Homopolymers

**Figure 10:**
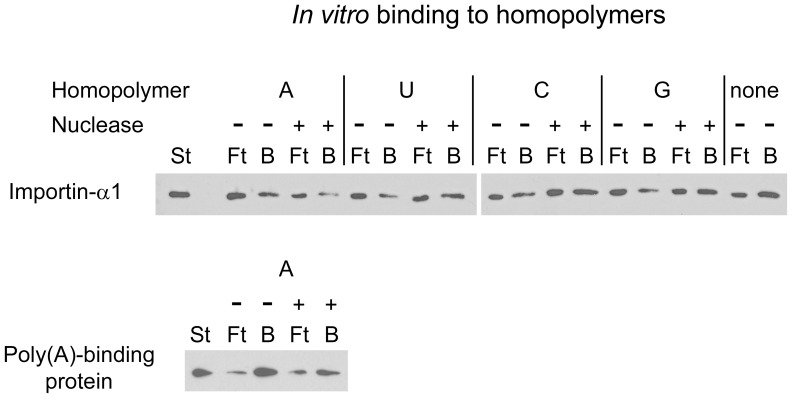
Interaction of purified importin-α1 with RNA homopolymers *in vitro.* His6-importin-α1 or His6-poly(A)-binding protein was synthesized in *E. coli*. Purified importin-α1 was incubated with immobilized poly(A), poly(U), poly(C) or poly(G) homopolymers or non-conjugated resin. In control experiments, resins were pre-treated with micrococcal nuclease as indicated. Aliquots of the flow through (10%, Ft) and bound material (100%, B) were analyzed by Western blotting with antibodies against importin-α1 (top). Under the same conditions, a strong interaction between immobilized poly(A) and purified poly(A)-binding protein was detected with antibodies against the His-tag (bottom).

Data described in Fig. 9 show that importin-α1 co-purified with poly(A)-RNA isolated from growing cells. One possible mechanism underlying this association is the direct binding of importin-α1 to RNA. A commonly used assay to detect such direct interactions relies on immobilized RNA homopolymers and proteins purified from *E. coli*. For example, this method was used to pull down purified poly(A)-binding protein *in vitro* [57]. Therefore, we included the interaction between poly(A)-binding protein and poly(A)-sepharose as a positive control that validated the assay (Fig. 10). Several negative controls monitored the non-specific interactions of importin-α1. First, each homopolymer-sepharose was pre-treated with micrococcal nuclease to reduce the number of binding sites provided by RNA. Second, purified importin-α1 was incubated with non-conjugated resin, i. e. resin that was not coupled to a homopolymer. For each sample, aliquots of the unbound (Ft) and bound protein (B) were probed by Western blotting with antibodies against importin-α1. With this assay, little or no difference of importin-α1 binding was detected when reactions were treated with or without nuclease (Fig. 10). This indicates that importin-α1 is unlikely to bind homopolymers directly. By contrast, purified poly(A)-binding protein associated efficiently with immobilized poly(A), and this interaction was diminished when the poly(A)-sepharose was pre-treated with micrococcal nuclease. Taken together, importin-α1 purified from *E. coli* exhibited no or only weak interactions with homopolymers *in vitro.*

## Discussion

Importin-α plays an essential role in nucleocytoplasmic transport by serving as an adaptor between the cNLS and importin-β1. One of the immediate responses to stress is nuclear transport inhibition. We have shown previously that heat shock and DEM interfere with nuclear import and export by affecting multiple transport factors [33], [34], [54]. Besides altering nuclear transport, stress also leads to translational arrest which is accompanied by the formation of SGs that contain RNA-binding proteins and poly(A)-RNA.

Our current work demonstrates that the oxidant DEM induces the formation of SGs that contain importin-α1. Furthermore, we show for the first time that importin-α4 and α5, members of the α3 and α1 subfamilies, are targeted to SGs as well. The SG composition is stress-specific [3], [58]; yet, importin-α1, α4 and α5 also accumulated in SGs that were induced by arsenite or heat shock. Thus, several importin-α proteins of distinct subfamilies are recruited to SGs under different stress conditions, and importin-α1, α4 and α5 can be regarded as *bona fide* SG constituents. Importantly, their association with SGs is specific, because they do not concentrate in PBs under normal or stress conditions (summarized in Table 1). This property is shared by importin-β1, but not CAS, both of which are carrier proteins that interact directly with importin-α family members.

**Table 1:** Summary of results for the association of nuclear transport factors with SGs and PBs.

Transport factor	Normal distribution	Stress	Presentin SG	Present in PB
Importin-α1	N+C; NE	N>C	yes	no
Importin-α4	N+C; NE	N>C	yes	no
Importin-α5	N+C; NE	N>C	yes	no
Importin-β1	N>C	N>C	yes	no
CAS	N>C	N>>C	no	no

Data for the stress-induced relocation of nuclear transport factors and their association with SGs or PBs are depicted.

Aside from its accumulation in SGs under different stress conditions, importin-α1 associated with poly(A)-RNA *in vivo*, and DEM significantly reduced this interaction. Interestingly, the RNA association of importin-α1 was a distinguishing feature of this isoform, which was not shared by any of the other transport adaptors analyzed here. Our binding assays suggest that importin-α1 synthesized in *E. coli* did not efficiently associate with RNA homopolymers *in vitro.* There are several potential explanations for this observation. First, it is possible importin-α1 does not contact RNA directly, and a linker protein is necessary to connect importin-α1 to poly(A)-RNA in growing cells. Second, posttranslational modifications of importin-α1 may be necessary for its binding to RNA, and these modifications are absent when importin-α1 is purified from *E. coli*. Third, importin-α1 recognizes specific RNA sequences that are not provided by homopolymers. Future studies will have to distinguish between these possibilities to determine the precise mechanisms that promote the interaction between importin-α1 and poly(A)-RNA *in vivo*.

Independent of the nature of its RNA-binding, the coincidence of importin-α1 release from poly(A)-RNA and SG association could suggest that the stress-induced dissociation of RNA/importin-α1 complexes is linked to SG recruitment. In support of this recruitment, SG components could provide binding sites for importin-α1; HuR is a potential candidate for this interaction, as it is an established binding partner of importin-α1 under nonstress conditions [59].

In an alternative model, nuclear transport factors may promote SG assembly by moving individual constituents to SGs. According to this idea, members of the importin-α family and importin-β1 will deliver material to the growing granule [48], a hypothesis compatible with the fact that importin-α1 knockdown reduces SG size [38]. Hence, nuclear transport factors may not only deliver macromolecules across nuclear membranes, but also to specialized cytoplasmic compartments, as they are exemplified by SGs. This model is further supported by the observation that transportin-1 participates in the movement of material between SGs and PBs, while importin-8 is involved in export from PBs [11], [60].

Collectively, our experiments provide novel insights into the biological roles of several members of the importin-α family, especially the multifunctional protein importin-α1. The newly identified ability of importin-α1 to associate with poly(A)-RNA is particularly interesting, because it is controlled by stress. Moreover, we identified additional members of the importin-α family, which belong to different subfamilies, as novel SG constituents. While these factors have an established role in nuclear protein transport or gene expression regulation [36], [47], our results suggest additional isoform-specific functions that are related to RNA metabolism and the stress response.

## Supporting Information

Figure S1 **Specificity of anti-importin-α antibodies for immunolocalization.** Primary antibodies against members of the importin-α family and isotype-specific IgG controls (for importin-α1 and α5) or pre-immuneserum (control for importin-α4) were tested under the same conditions. Staining was evaluated for ethanol and DEM-treated cells as described for Fig. 1. All samples were co-stained with antibodies against HuR, and nuclei were detected with DAPI. Size bar is 20 µm.
(TIF)

Figure S2 **Western blot analysis determines the specificity of antibodies against nuclear transport factors and HuR.** Crude HeLa cell extracts were tested with antibodies against nuclear transport factors, HuR or control antibodies as indicated. Negative control antibodies, either isotype-specific IgG or pre-immuneserum (PS), were used at the same concentration as primary antibodies. For each antigen, the same filter was probed with primary and control antibodies, with identical exposure times during ECL.
(TIF)
