# Supplementary figures and images for: Identification of Novel Stress Granule Components That Are Involved in Nuclear Transport
Source: PLoS One. 2013 Jun 27;8(6):e68356. doi: 10.1371/journal.pone.0068356 (PMC3694919; doi:10.1371/journal.pone.0068356)

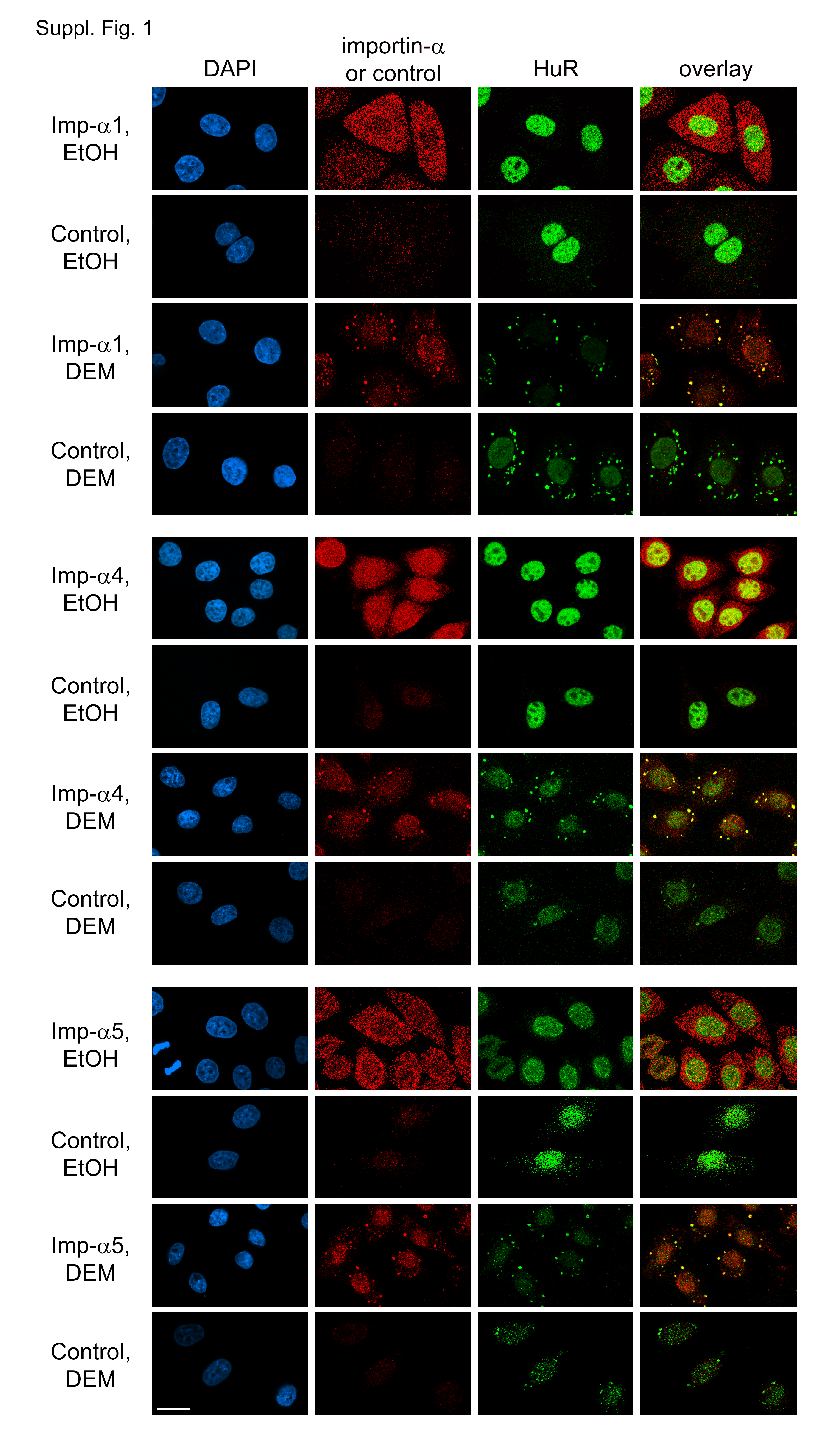

Supplement: Figure S1 — Specificity of anti-importin-α antibodies for immunolocalization. Primary antibodies against members of the importin-α family and isotype-specific IgG controls (for importin-α1 and α5) or pre-immuneserum (control for importin-α4) were tested under the same conditions. Staining was evaluated for ethanol and DEM-treated cells as described for Fig. 1. All samples were co-stained with antibodies against HuR, and nuclei were detected with DAPI. Size bar is 20 µm. (TIF) [file pone.0068356.s001.tif]

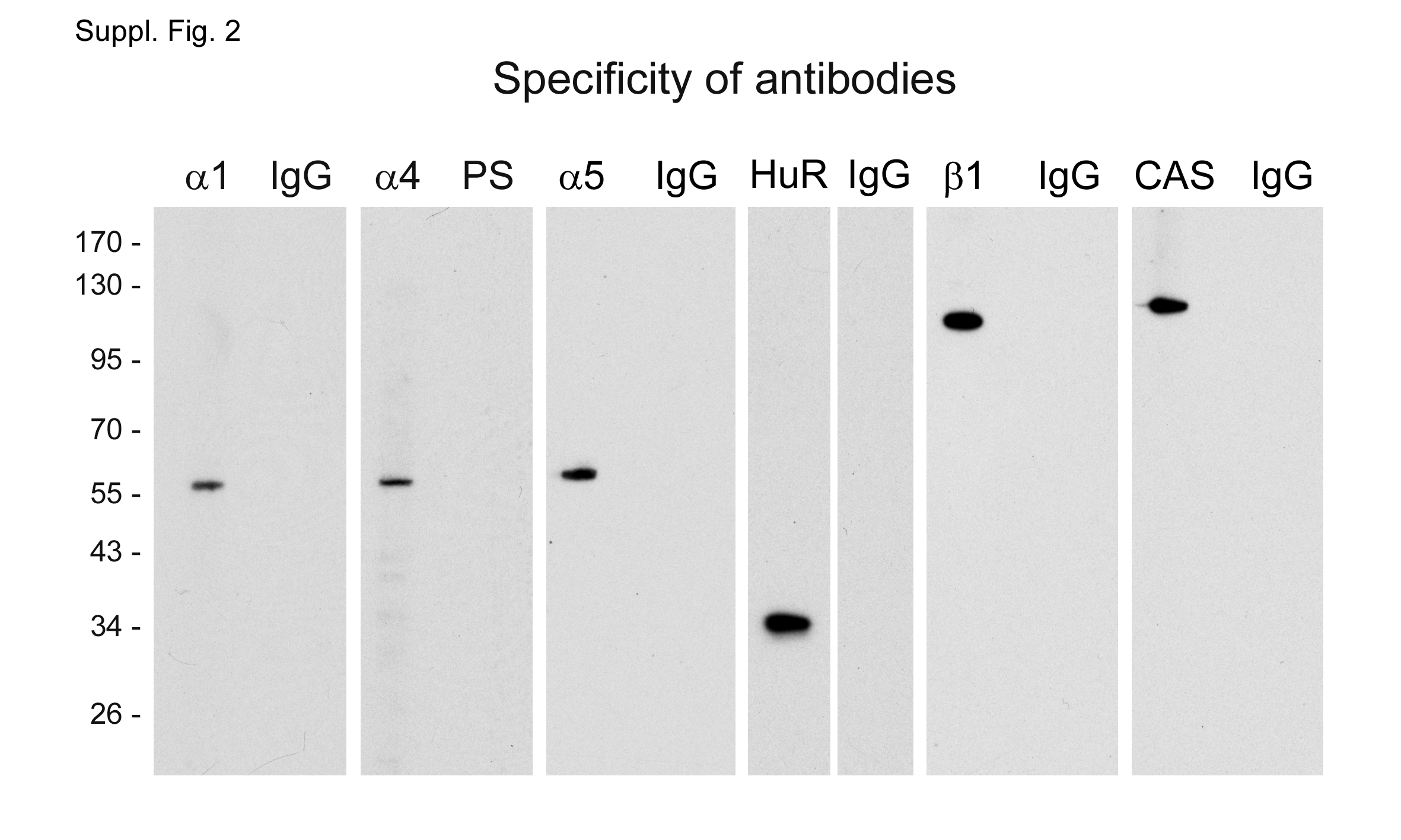

Supplement: Figure S2 — Western blot analysis determines the specificity of antibodies against nuclear transport factors and HuR. Crude HeLa cell extracts were tested with antibodies against nuclear transport factors, HuR or control antibodies as indicated. Negative control antibodies, either isotype-specific IgG or pre-immuneserum (PS), were used at the same concentration as primary antibodies. For each antigen, the same filter was probed with primary and control antibodies, with identical exposure times during ECL. (TIF) [file pone.0068356.s002.tif]
